# Efficacy of Intraperitoneal 0.2% Ropivacaine With Dexmedetomidine Versus 0.2% Ropivacaine With Ketamine in Laparoscopic Surgeries: A Randomized Controlled Trial

**DOI:** 10.7759/cureus.38035

**Published:** 2023-04-24

**Authors:** Amrita Panda, Mousumi Das, D Dhatri, Ganesh C Satapathy

**Affiliations:** 1 Anesthesiology, Kalinga Institute of Medical Sciences, Bhubaneswar, IND

**Keywords:** intraperitoneal, analgesic efficacy, laparoscopic surgery, adjuvants, local anaesthetics

## Abstract

Background: Effective pain management modalities are the armamentarium for enhanced recovery in laparoscopic surgeries. Intraperitoneal instillation of local anaesthetics with adjuvants is advantageous in minimizing pain. So, we designed this study with the aim to compare the analgesic effectiveness of intraperitoneal ropivacaine with adjuvants like dexmedetomidine versus ketamine for postoperative analgesia.

Objective: The objective of this study is to assess the total duration of analgesia and total rescue analgesic dose requirements in the first 24 hours postoperatively.

Materials and methods: A total of 105 consenting patients for elective laparoscopic surgeries were enrolled and divided into three groups by computer-generated randomization as follows: Group 1: 30 ml of 0.2% ropivacaine with ketamine 0.5 mg/kg diluted to 1 ml; Group 2: 30 ml of 0.2% ropivacaine with dexmedetomidine 0.5 mcg/kg diluted to 1 ml; Group 3: 30 ml of 0.2% ropivacaine with 1 ml of normal saline. The postoperative visual analogue scale (VAS) score, total duration of analgesia, and total analgesic dose were calculated and compared among the three groups.

Results: The postoperative analgesic duration after intraperitoneal instillation of Group 2 was longer as compared to Group 1. The total analgesic requirement was lower in Group 2 as compared to Group 1, and the p-value was significant (p ≤ 0.001) for both parameters. Demographic parameters and VAS scores among the three groups were not statistically significant.

Conclusion: We conclude that intraperitoneal instillation of local anaesthetics with adjuvants is effective for postoperative analgesia in laparoscopic surgeries, and ropivacaine 0.2% with dexmedetomidine 0.5 mcg/kg is more effective when compared to ropivacaine 0.2% with ketamine 0.5 mg/kg.

## Introduction

Laparoscopy aims to achieve the therapeutic goals of surgery with minimal somatic and physiological trauma. Visceral pain in these patients delays a speedy recovery and poses a challenge for enhanced recovery after surgery [1]. This visceral signalling is through the enteric nervous system (the myenteric (Auerbach) and the submucosal (Meissner) plexuses) and is maximally observed during the first hour of the postoperative period [2].

Intraperitoneal instillation of local anaesthetics is an effective mode of postoperative analgesia in laparoscopic surgeries amongst the various multimodal analgesia approaches. Visceral nociceptive receptors on the exposed peritoneum are blocked, thereby aiding analgesia. Systemic absorption also plays a role in reduced nociception [3].

Adjuvants added to local anaesthetics have proved to be of additional advantage in minimizing pain due to their antinociceptive mechanism of action.

Dexmedetomidine being α-2 adrenergic agonist is a widely used anaesthetic drug due to its opioid-sparing effect, sedation, and analgesic property. It prolongs the duration of local anaesthetic action [4]. Ketamine being an N-methyl-D-aspartate (NMDA) receptor antagonist has hypnotic and analgesic activity, so it enhances the analgesic activity of local anaesthetics [5].

Aim

The current study aims to compare the analgesic efficacy of intraperitoneal ropivacaine with adjuvants like dexmedetomidine versus ketamine for postoperative analgesia in laparoscopic surgeries.

Primary objective

The primary objective of this study is to assess the total duration of analgesia (the time duration from the completion of surgery to the time patient requested for first rescue analgesic medication) and total rescue analgesic dose requirements in the first 24 hours postoperatively.

Secondary objective

The secondary objectives of the study are as follows: (1) to compare the intensity of pain using the visual analogue scale (VAS) score every 30 minutes for the first two hours and then after six hours, 12 hours, and 24 hours postoperatively; (2) to compare the haemodynamic parameters (mean arterial pressure (MAP) and heart rate (HR)) intraoperatively at 0 minutes, 15 minutes, 30 minutes, 60 minutes, and 90 minutes and postoperatively every 30 minutes for the first two hours and then after six hours, 12 hours, and 24 hours; (3) to study the incidence of postoperative nausea and vomiting and shoulder tip pain after laparoscopic surgery.

## Materials and methods

Informed and written consent was obtained from all participating subjects. This article has been prepared according to the Consolidated Standards of Reporting Trials guidelines.

This study was conducted at Pradyumna Bal Memorial Hospital, Kalinga Institute of Medical Sciences, Odisha, India, from January 2021 to March 2022. Each of the consenting participants aged between 18 and 60 years, scheduled to undergo elective laparoscopic surgery under general anaesthesia, belonging to the American Society of Anesthesiologists (ASA) physical status I and II was enrolled. Patients with a history of allergy to local anaesthetics or study drugs, pregnancy, and patients with any other systemic illness such as cardiovascular and pulmonary diseases limiting physical activity were excluded.

Randomization was done by computer-generated randomization tables. After considering the inclusion criteria, random allocation of patients was done to one of the three groups by means of an opaque sealed envelope technique to reduce the chance of bias. A double-blind technique was followed, where the patient and the primary investigator were unaware of the drug regimen. An anesthesiologist who was not part of the study prepared the drug according to the allocated group.

Study groups

Group 1: 30 ml of 0.2% ropivacaine with ketamine 0.5 mg/kg diluted to 1 ml. Group 2: 30 ml 0.2% ropivacaine with dexmedetomidine 0.5 mcg/kg diluted to 1 ml. Group 3: 30 ml of 0.2% ropivacaine with 1 ml of normal saline.

Procedure

After a discussion of the risks and benefits, informed consent was obtained. Preoperative evaluation and assessment were done. The patient was transferred into the operating room. After connecting standard monitors (electrocardiogram, oxygen saturation, non-invasive blood pressure, and capnography), basal haemodynamic parameters were noted. Premedication was done with intravenous glycopyrrolate 0.2 mg, midazolam 1 mg, and nalbuphine 0.1 mg/kg. Preoxygenation was performed for three minutes with 100% oxygen and bag-mask ventilation. Induction was accomplished with propofol 2 mg/kg, followed by succinylcholine (1.5 mg/kg) and intubation was performed with an appropriate-size endotracheal tube. Maintenance of anaesthesia was performed with nitrous oxide and oxygen mixture (1:1) and isoflurane at 1 MAC. Intraoperative neuromuscular blockade was maintained with vecuronium. During the surgery, end-tidal carbon dioxide was maintained between 35 and 45 mm of Hg.

**Figure 1:**
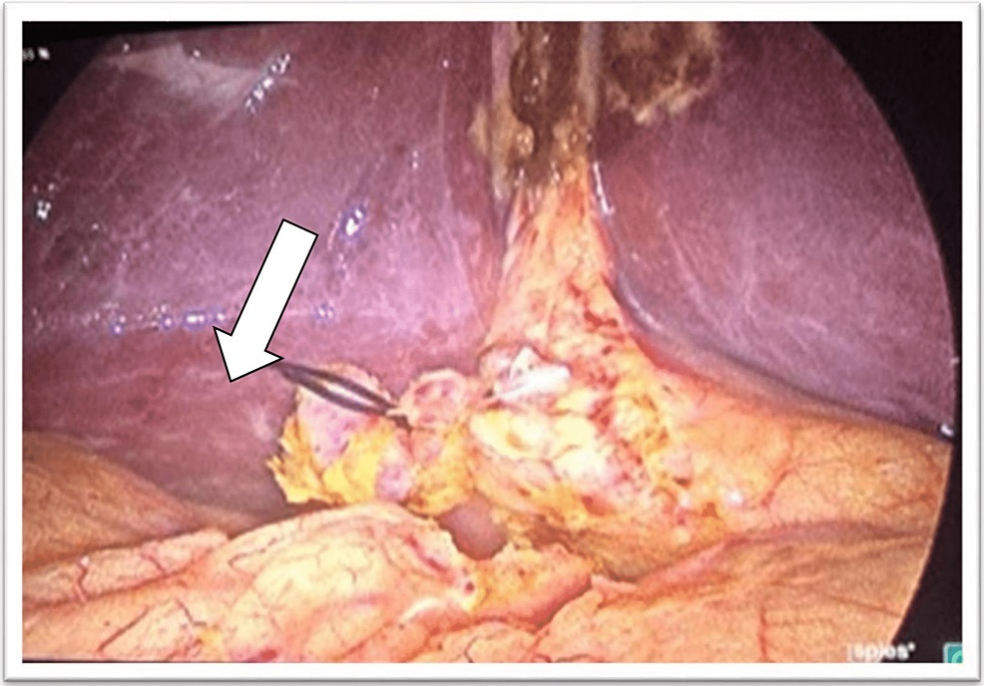
Intraperitoneal drug instillation

Following the skin incision, an 11 mm trocar was inserted through the umbilical port and a pneumoperitoneum was created to maintain intra-abdominal pressure between 12 and 14 mmHg. After the surgery, 30 ml of 0.2% ropivacaine with 1 ml of adjuvant was instilled intraperitoneally at the surgical site and below the two cupulas of the diaphragm under vision using a trocar, as shown in Figure 1. A Trendelenburg position was maintained for five minutes after instillation. Paracetamol IV 15 mg/kg was given intraoperatively towards the end of surgery.

After the removal of the trocar, the pneumoperitoneum was evacuated completely and the wound was closed in layers. After ensuring adequate spontaneous ventilation and neuromuscular recovery, the trachea was extubated. All the patients were shifted to post anaesthesia care unit for observation. Primary and secondary outcomes were measured. VAS score was noted at different time intervals. Tramadol IV 1 mg/kg was given as the rescue analgesia whenever the VAS score was more than 4.

The time for the first rescue analgesic requirement and total analgesic dose requirement for 24 hours was noted. The presence of any complications was noted.

Outcome measures

The primary outcome of the study was to assess the total duration of analgesia in hours (the time duration from the completion of surgery to the time patient requested the first analgesic medication) and the total rescue analgesic dose requirements (in mg) in the first 24 hours postoperatively.

The secondary outcomes included the following: (1) to compare the intensity of pain using VAS score postoperatively every 30 minutes for two hours and then after six, 12, and 24 hours; (2) to compare the haemodynamic parameters (mean arterial pressure in mm of Hg and heart rate in beats per minute) intraoperatively at 0 minutes, 15 minutes, 30 minutes, 60 minutes, and 90 minutes and postoperatively every 30 minutes for the first two hours and then after six hours, 12 hours, and 24 hours; (3) to study the incidence of postoperative nausea and vomiting using the Likert scale and shoulder tip pain after laparoscopic surgery using the VAS score.

The outcomes were recorded by an observer who was blinded to the group allocation.

Statistical analysis

The continuous variables were expressed by mean ± standard deviation and the categorical variables were expressed by frequency and percentage. The data have undergone a normality test by the Shapiro-Wilk test. Student's t-test was done for comparison of two groups. The chi-square test was done to check the association between two categorical variables. Repeated measure ANOVA was performed to check the effect of haemodynamic parameters over a time period and to compare between three groups. A p-value < 0.05 was considered statistically significant. Statistical analysis was done by using SPSS software version 23 (IBM Corp., Armonk, NY).

Sample size collection

The sample size was calculated in reference to a previous study by Alireza Kamali et al., in which patients were divided into two groups, one received dexmedetomidine (1 mg/kg in 100 ml normal saline) and the other received ketamine (5 mg/kg in 100 ml normal saline) intraperitoneally after caesarean section. The total duration of analgesia (in hours) in the two groups was 12.8 ± 2.8 and 5.1 ± 13.3. The minimum required sample size wascalculated using SPSS version 23, with a 5% level of significance and 90% power and an effect size of 0.78, i.e., 35 in each group [6]. Additionally, a control group with the same number of participants has been included in this study.

## Results

A total of 105 participants who fulfilled the eligibility criteria were randomized into three groups and included in the study. Demographic characteristics were comparable among the three groups (Table 1) and no significant difference was observed.

**Table 1:** Comparison of demographic data, American Society of Anesthesiologists status, height, weight, and BMI among the three groups ASA PS: American Society of Anesthesiologists physical status.

Demographic parameters	Group 1 (N = 35)	Group 2 (N = 35)	Group 3 (N = 35)	P-value
Age (years)	39.89 ± 11.972	40.41 ± 12.481	39.23 ± 13.137	0.926
Height (cm)	167.60 ± 7.196	163.75 ± 6.205	164.03 ± 8.219	0.052
Weight (kg)	68.26 ± 8.240	66.00 ± 8.087	65.11 ± 8.646	0.271
BMI (kg/m^2^)	24.337 ± 2.9633	24.779 ± 3.6210	24.291 ± 3.5726	0.806
Sex, female	14	18	19	0.281
Sex, male	22	16	16	
ASA PS I	20	19	19	0.971
ASA PS II	15	15	17	

**Figure 2:**
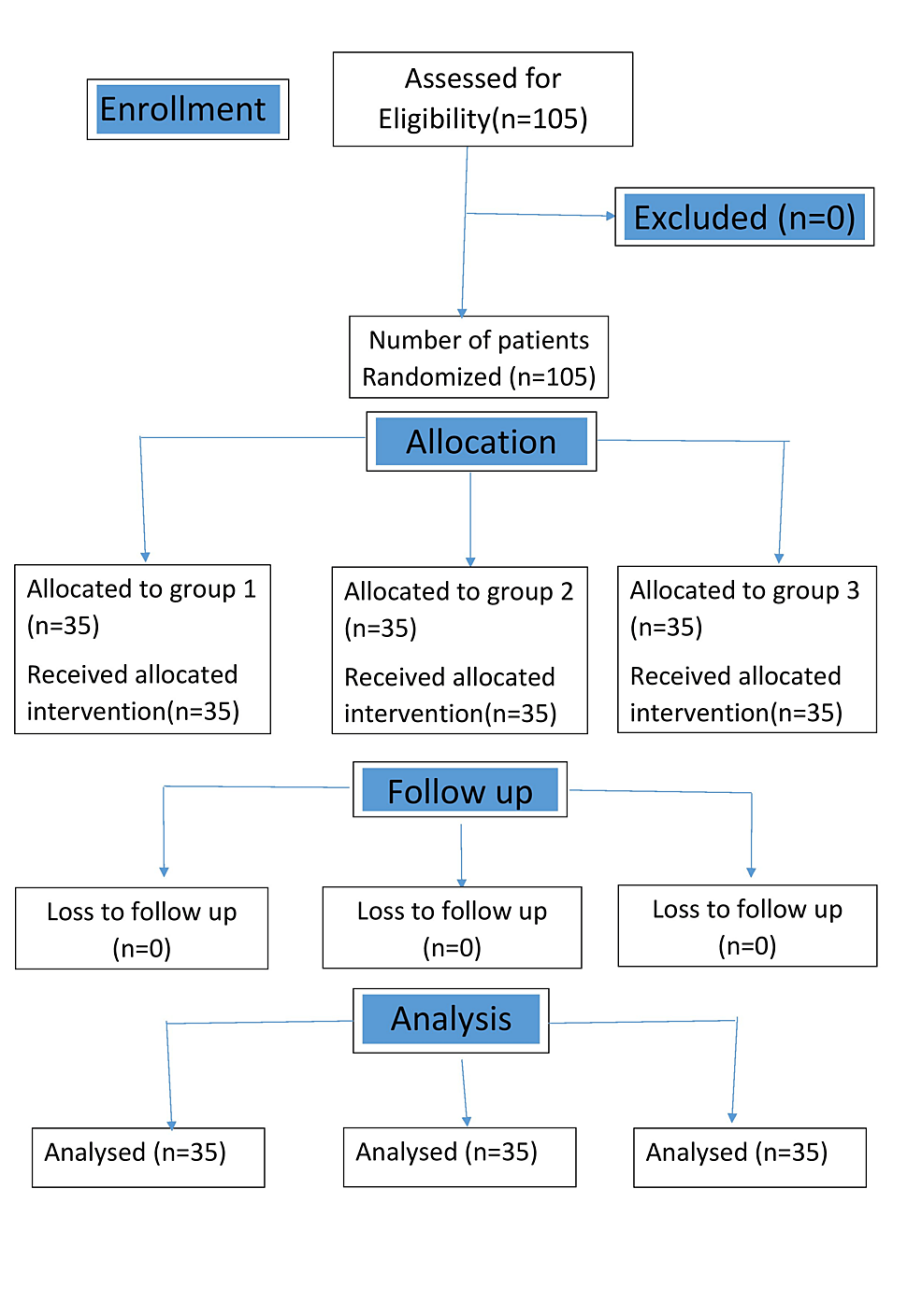
Consolidated Standards of Reporting Trials (CONSORT) diagram

The flowchart of the study is represented in Figure 2.

The primary outcomes of the study were comparable among the three groups (Table 2).

**Table 2:** Primary outcomes of the study

Parameter	Group 1 (N = 35)	Group 2 (N = 35)	Group 3 (N = 35)	P-value
Duration of analgesia (hours)	4.614 ± 1.3122	5.059 ± 1.5313	3.414 ± 1.2217	<0.001
Total dose of tramadol received in 24 hours (mg)	118.29 ± 17.903	97.35 ± 30.482	151.43 ± 31.45	<0.001

Group 1 had a mean analgesic time of 4.614 ± 1.3122 hours, Group 2 had a mean analgesic time of 5.059 ± 1.5313 hours, and Group 3 had a mean analgesic time of 3.414 ± 1.2217 hours. There was a highly significant statistical difference in mean analgesic time among the three groups with a p-value < 0.001.

Group 1 had a total analgesic dose of 118.29 ± 17.903 mg, Group 2 had a total analgesic dose of 97.35 ± 30.482 mg, and Group 3 had a total analgesic dose of 151.43 ± 31.450 mg. The analysis showed a highly statistically significant difference in the total dose of analgesia among the three groups with a p-value < 0.001. Intraperitoneal instillation of local anaesthetics provided postoperative analgesia in laparoscopic surgeries. Adding adjuvants to local anaesthetics prolonged the duration of analgesia thereby sparing opioid use in the postoperative period.

The static and dynamic VAS scores at different time intervals in the postoperative period were compared among the three groups. No statistical significance was found in VAS scores.

Intraoperative and postoperative haemodynamic parameters were compared and no statistically significant difference was noticed among the three groups.

Table 3 shows the intraoperative mean arterial pressure readings at different time intervals. No significant difference was observed among the three groups in terms of mean arterial pressure.

**Table 3:** Mean arterial pressure intraoperatively

Mean arterial blood pressure (MAP) measured at different time periods	Group 1 (N = 35)	Group 2 (N = 35)	Group 3 (N = 35)	P-value		
					0 min	92.235 ± 8.4210	89.618 ± 10.3544	87.200 ± 12.4683	0.146		
15 min	90.829 ± 11.7410	95.029 ± 9.1104	90.771 ± 10.8982	0.170		
30 min	90.000 ± 10.0264	92.441 ± 10.6407	91.543 ± 10.8583	0.622		
60 min	86.629 ± 11.6242	90.441 ± 11.5659	92.371 ± 12.8363	0.132		
90 min	93.912 ± 8.5080	93.735 ± 6.3020	91.941 ± 9.7388	0.558		

Table 4 shows the heart rate intraoperatively at different time periods. This shows a significant difference in heart rate at 60 minutes with a p-value of 0.011 due to the lighter plane of anaesthesia.

**Table 4:** Heart rate intraoperatively

Heart rate (HR) measured at different time periods	Group 1 (N = 35)	Group 2 (N = 35)	Group 3 (N = 35)	P-value		
					0 min	85.571 ± 10.6060	84.471 ± 11.5135	81.943 ± 10.3666	0.360		
15 min	85.200 ± 11.8192	87.088 ± 9.6023	82.086 ± 9.9982	0.142		
30 min	82.800 ± 11.0475	87.265 ± 9.1231	83.571 ± 9.3373	0.140		
60 min	81.200 ± 9.8989	82.588 ± 9.7704	87.971 ± 9.5655	0.011		
90 min	85.441 ± 9.9520	84.412 ± 9.9335	85.294 ± 9.9743	0.899		

Table 5 shows the postoperative mean arterial pressure readings at different time intervals. A significant difference was observed at 60 minutes postoperatively with a p-value of 0.047, which can be a post-extubation response or associated physiology of the patient.

**Table 5:** Postoperative mean arterial pressure among the three groups

Mean arterial pressure (MAP) measured at different time periods	Group 1 (N = 35)	Group 2 (N = 35)	Group 3 (N = 35)	P-value		
					0 min	96.20 ± 12.070	96.29 ± 6.571	93.97 ± 8.137	0.497		
30 min	93.20 ± 9.932	92.68 ± 8.626	92.31 ± 10.789	0.931		
60 min	90.37 ± 9.768	94.88 ± 7.784	95.34 ± 9.643	0.047		
90 min	90.00 ± 10.398	91.18 ± 9.919	90.00 ± 10.381	0.860		
120 min	92.74 ± 7.694	91.47 ± 8.450	94.66 ± 8.731	0.280		
6 hours	93.74 ± 10.251	90.06 ± 7.160	91.71 ± 10.445	0.271		
12 hours	92.40 ± 8.852	93.12 ± 8.134	92.74 ± 8.343	0.940		
24 hours	89.46 ± 8.672	91.59 ± 7.913	92.37 ± 9.453	0.354		

Table 6 shows the postoperative heart rate readings at different time intervals. No significant difference was observed among the three groups in terms of heart rate.

**Table 6:** Postoperative heart rate among the three groups

Heart rate (HR) measured at different time periods	Group 1 (N = 35)	Group 2 (N = 35)	Group 3 (N = 35)	P-value		
					0 min	84.77 ± 10.795	85.71 ± 10.238	86.89 ± 10.802	0.707		
30 min	85.23 ± 9.900	85.53 ± 10.145	82.77 ± 10.114	0.458		
60 min	85.91 ± 10.115	83.71 ± 10.059	85.63 ± 9.506	0.605		
90 min	83.09 ± 11.518	85.12 ± 10.542	86.49 ± 10.371	0.420		
120 min	87.74 ± 10.934	85.32 ± 9.098	85.03 ± 11.501	0.503		
6 hours	86.11 ± 10.459	87.32 ± 11.862	83.31 ± 11.417	0.319		
12 hours	86.06 ± 10.085	83.85 ± 12.270	88.00 ± 10.163	0.289		
24 hours	83.83 ± 10.422	85.00 ± 11.410	87.40 ± 10.508	0.373		

## Discussion

Laparoscopic procedures are gaining popularity in recent years because of minimal incisions, brief hospital stays, and early ambulation. The main reason for prolonged hospital stay after surgery is pain. Perception of pain is different for different individuals and causes haemodynamic alterations in patients [7]. This acute pain being complex in nature suggests that postoperative analgesia should be multimodal. Various multimodal analgesia techniques were studied for providing pain relief like performing surgery under the subarachnoid block, epidural infusion, parenteral opioids and non-steroidal anti-inflammatory drugs, and intraperitoneal instillation of local anaesthetics. Intraperitoneal instillation of local anaesthetics is a simple and effective method as a part of multimodal analgesia.

Visceral pain in laparoscopic surgeries occurs due to the rapid creation of pneumoperitoneum, intraperitoneal inflammation, diaphragmatic irritation, and vascular and neural traction. Viscera convey autonomic reactions and unpleasant sensations through the vagus nerve [2]. These nociceptors, which are activated by intraperitoneal inflammation, influence illness and feeding behaviours. Intraperitoneal instillation of local anaesthetic blocks these visceral nociceptors [8].

**Figure 3:**
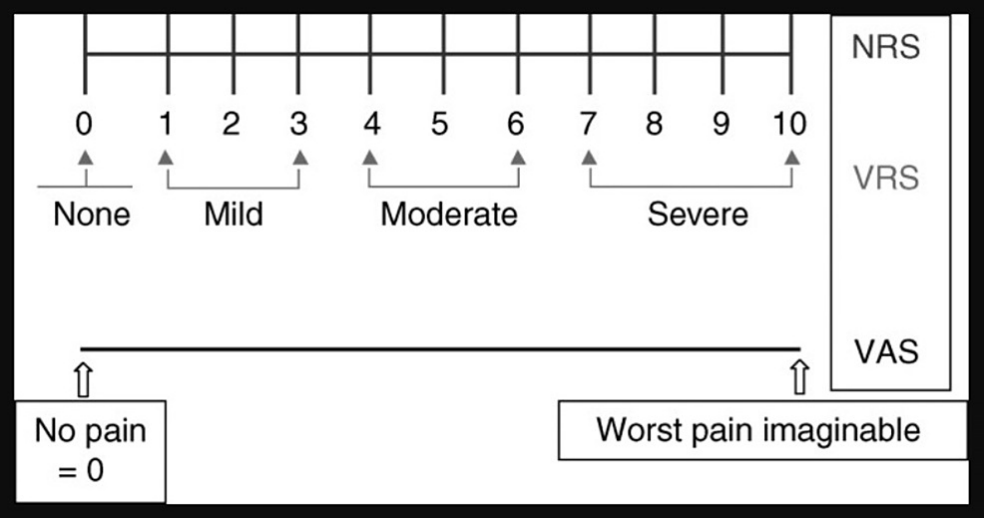
Visual analogue scale (VAS) NRS: numeric rating scale; VRS: verbal rating scale. Adapted from: Breivik H, Borchgrevink PC, Allen SM, et al. Assessment of pain. Br J Anaesth. 2008;101(1):17-24. doi: 10.1093/bja/aen103.

As pain is uniquely subjective in nature with no objective signs, we have to accept individuals' “self-report” of the severity of the pain they are experiencing. This pain was estimated by VAS score. VAS score employs a 10 cm line drawn with the left anchor point descriptor labelled “No pain” and the right-sided equivalent labelled “Worst possible pain” (Figure 3).

The rescue analgesic used in our study was intravenous tramadol in a dose of 1 mg/kg body weight diluted in 100 ml of normal saline when the VAS score as described by the patient was more than 4. In our study, it was found that the postoperative dose of rescue analgesic for 24 hours was significantly less in ropivacaine with dexmedetomidine group (97.35 ± 30.482 mg) as compared to the ropivacaine with ketamine group (118.29 ± 17.903 mg). The control group received much higher doses of opioids (151.43 ± 31.450 mg) as compared to the other two groups. In a past study, the total dose of fentanyl required in the intraperitoneal ropivacaine group was less when compared to the control group [9]. This was statistically significant with a p-value of <0.001 and strongly correlated with our study.

The timing of the instillation of local anaesthetic intraperitoneally and the appropriate method of instillation are of utmost importance. Unlike bupivacaine used in previous studies, we preferred ropivacaine because of its better safety profile with minimal side effects. The vaso-constricting property of ropivacaine prevents the systemic absorption of the drug and hence avoids cardiac and neuronal complications. However, surgically dissected wounds after laparoscopic surgery may increase the absorption of ropivacaine.

A study was conducted where the total amount of fentanyl consumed was lower in patients receiving intraperitoneal ropivacaine as compared with the placebo group. This correlated well with our current study where the rescue analgesic requirement was less after intraperitoneal instillation of ropivacaine [10].

Adding an adjuvant with a lower concentration of local anaesthetics will produce analgesia that is comparable to that produced by the higher concentration of anaesthetics alone. We used dexmedetomidine and ketamine as adjuvants to ropivacaine to compare the analgesic efficacy.

In our study, the time taken for the first rescue analgesic was compared among the three groups. Patients in the ropivacaine with dexmedetomidine group had a longer duration of analgesia (in hours) postoperatively (5.059 ± 1.5313) as compared to ropivacaine with ketamine group (4.614 ± 1.3122). Patients in the placebo group had a shorter duration of analgesia as compared to the other two groups of subjects (3.414 ± 1.2217).

In another study in which patients received 50 ml of 0.25% bupivacaine and 50 ml of 0.25% bupivacaine with dexmedetomidine 1 mcg/kg after laparoscopic surgeries, bupivacaine with dexmedetomidine provided longer analgesic duration postoperatively as compared with bupivacaine alone [11]. This correlates with our present study that adding an adjuvant can prolong the duration of analgesia.

A previous study demonstrated that intraperitoneal ketamine can be used for postoperative analgesia after laparoscopic cholecystectomy. Ketamine is a non-competitive NMDA antagonist with an analgesic effect [12]. It is absorbed easily by crossing tissue membranes.

In our study, no statistical difference was observed in VAS scores at different time periods in the postoperative period among the three groups. A past study compared 0.125% bupivacaine with normal saline intraperitoneally [13]. The mean VAS score for the bupivacaine group in the second hour was 2 and for the saline group was 6. The mean VAS score for bupivacaine in the fourth hour was 2 and for the saline group was 4, which does not correlate with our study.

In a study conducted previously, ropivacaine 0.5% and saline were both administered intraperitoneally, close to the gall bladder fossa [13]. Patients receiving ropivacaine in the postoperative phase had better VAS scores and improved pain alleviation up until the fourth postoperative hour as compared to the saline group. This is consistent with our research.

A study compared bupivacaine 100 mg with ropivacaine 150 mg for intraperitoneal instillation. Both groups had low pain scores and no significant variation in haemodynamic parameters was observed, which was similar to our study [14].

In our study, we found a significant rise in heart rate intraoperatively at 60 minutes due to a lighter plane of anaesthesia (Table 4). Also, there was a significance of systolic blood pressure postoperatively at 0 minutes after extubation. The same can be explained for the significance found in mean arterial pressure at 60 minutes postoperatively (Table 5).

No significant complications like haemodynamic compromise or systemic toxicity were noted in our study. There were no side effects like postoperative nausea and vomiting and shoulder tip pain.

Limitations of the study

This study is a single-centre study. The study had a small sample size and the follow-up period was limited to 24 hours postoperatively. The time taken for extubation was not measured intraoperatively. Laparoscopic port site infiltration was not done. Hence, somatic pain was not evaluated and VAS scores were not significant.

## Conclusions

We conclude that intraperitoneal instillation of local anaesthetic drugs is an effective mode of postoperative analgesia in patients undergoing laparoscopic surgeries. Adding an adjuvant provides the benefit of prolonged postoperative analgesia with well-maintained haemodynamic parameters. Dexmedetomidine as an adjuvant to local anaesthetic drugs significantly increases the duration of postoperative analgesia as compared to ketamine.
